# An evaluation of a morphine public health programme for cancer and AIDS pain relief in Sub-Saharan Africa

**DOI:** 10.1186/1471-2458-5-82

**Published:** 2005-08-10

**Authors:** Dorothy E Logie, Richard Harding

**Affiliations:** 1 Cheviot View, Bowden, Melrose, UK, TD6 OST; 2 Department of Palliative Care & Policy, Guy's King's & St Thomas' School of Medicine King's College London UK

## Abstract

**Background:**

Despite growing HIV and cancer prevalence in Sub-Saharan Africa, and WHO advocacy for a public health approach to palliative care provision, opioid availability is severely limited. Uganda has achieved a morphine roll-out programme in partnership with the Ministry of Health. This study aimed to evaluate that programme by identifying challenges to implementation that may inform replication.

**Methods:**

A multi-methods protocol appraised morphine regulation, storage, prescribing, and consumption in three phases: key informant interviews throughout the opioid supply chain, and direct observation and audit of clinical practice.

**Results:**

Regulation had achieved its goal of preventing misuse and leakage from the supply chain. However, the Government felt that relaxation of regulation was now appropriate. Confusion and complexity in storage and authorisation rules led to discontinuation of opioid pain management at the patient level and also wasted service time in trying to obtain supplies to which they were entitled. Continued neglect to prescribe among clinicians and public fear of opioids led to under prescribing, and clinical skills showed some evidence of need for improvement with respect to physical assessment and follow-up.

**Conclusion:**

The Ugandan programme offers a successful model for both advocacy and Governmental support in achieving opioid roll-out across health districts. Despite initial concerns, abuse of opioids has not been evident. Further work is required to ensure that available supplies of opioids are prescribed to those in need, and that clinical standards are met. However, the programme for roll-out has proved a useful model to expand opioid availability as the first step in improving patient care, and may prove a useful template for other Sub-Saharan African countries.

## Background

Most, if not all, pain due to cancer and AIDS could be relieved if we implemented existing medical knowledge [1]. This does not happen for two thirds of the world's population for several reasons. Most live in countries with weak public health infrastructure and, despite WHO encouragement for a public health approach to pain management and palliative care [2], a lack of legislative and policy support exists. Despite an HIV/AIDS epidemic which kills 3 million each year, inadequate availability of pain medication, especially of opioids, is evident in that only eleven out of forty seven African countries use morphine for chronic pain and, of these eleven, the amount consumed is tiny [3]. South Africa is highest user on the continent consuming 265 daily defined doses (DDD) while Namibia uses 97 and Uganda 9 DDDs, and eighty-six percent of the world's morphine is still used by the 20 richest countries [4]. Sadly, although morphine is a safe drug which can be used with anti-retroviral therapy, chemotherapy and traditional medicines, it is under-used largely due to professional fears.

Uganda is the first African country to follow the WHO guidelines, and, despite limited resources and a population which is 90% rural, has prioritised palliative care under Essential Clinical Services in the National Health Plan (2001–2005). It has made oral morphine freely available to those districts that have specialist palliative care nurses or clinical officers and has promoted morphine use down to village level. It has passed laws to allow nurse prescribing of morphine [5], an essential step as doctors are scarce in rural areas. There are no limits to the number of days, nor dose, which doctors or trained nurses and clinical officers can prescribe within the hospice setting, though only the weaker strength (5 mgs per 5 mls) was available to community nurses outside the Hospice setting.

Since 1993, Hospice Africa Uganda (HAU), a non-governmental organisation, has pioneered three community- based palliative care programmes in rural and urban localities. However, coverage has been comparatively low. In 1998, following several years of lobbying, the Ministry of Health invited hospice staff to be technical experts in a pilot study in 15 (out of 56) rural and urban districts to assess the feasibility and safety of using morphine for chronic pain in the community. This included cancer pain, pain from HIV/AIDS disease, and pain from sickle cell crisis. In 2002–03, the 15 districts, including mission hospitals, underwent extensive initial training involving local dignitaries, police, and senior health officials. The programme is still in the early stages and has faced challenges in funding, staffing and supervision. This paper reports an evaluation of the Ugandan morphine access programme, which aimed to appraise the processes of morphine supply, to assess clinical practice on prescribing and pain control, and investigate patient satisfaction with symptom control and costs. This initial outline of a working model of morphine access, achieved through policy, legislative and clinical efforts, was developed as an example for the continent. The strengths and blockages in the system of morphine delivery are described.

## Methods

### Setting

The study was undertaken in 2003 across rural and urban Ugandan hospice sites, two of the pilot districts Government District Hospitals, the Ministry of Health, and 2 home-based care NGO programmes. Ethics permission was obtained from the Ethics Committee of Liverpool School of Tropical Medicine and from Hospice Africa Uganda. Permission from participants was obtained by reading a standard permission sheet which explained about the survey, that it was confidential, voluntary, and would report anonymously. This was translated into local languages

### Procedure

The evaluation protocol employed mixed data collection methods from stakeholders throughout the supply chain.

Phase 1: semi-structured interviews among three populations. Firstly, clinicians prescribing morphine (n = 16); secondly, patients accessing morphine (n = 10); thirdly, key informants (n = 16) including senior clinical and Governmental staff. Interviews were voluntary and confidentiality was assured. Translation for patients was provided. Interviews were tape recorded, transcribed verbatim and thematically coded, and themes developed adopting framework analysis [6] using Winmax software.

Phase 2: direct observation of morphine from entry to the country to a pharmaceutical NGO and on to distribution through hospital wards and patients' homes. Pharmacy, ward and nursing records were checked against legal requirements [7].

Phase 3: two quality of clinical care audits across two hospice sites, urban and rural. The first audit observed domiciliary nurse visits prescribing oral morphine (n = 21), the second retrospectively examined prescribing standards in randomly-sampled clinical notes (n = 50). Criteria were established using local prescribing guidelines and clinical best-practice literature [8-10]. The random files sample was generated by selecting every 5^th ^deceased patient file for deaths recorded during a 12-month period 2002–2003.

## Results

**Figure 1:**
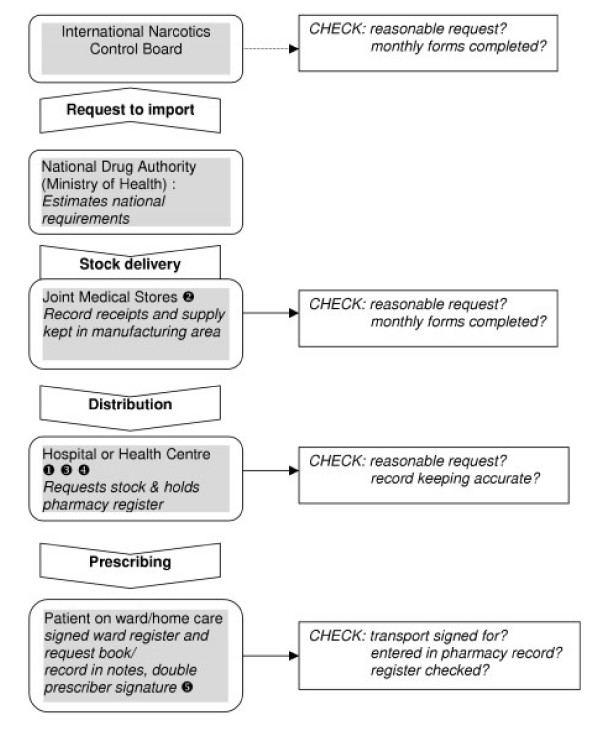
Morphine supply and blockages within the Ugandan regulatory framework. Blockages in morphine flow: key. *District Hospital *has to obtain permission from MOH for every order and take this to JMS. *National Medical Stores *currently re-structuring and lacks capacity and drug inspectors. *Mission hospitals and NGOs *have to obtain morphine via District Hospital pharmacy and depend on the co-operation of the pharmacist/dispenser. *Pharmacy *may be slow in re-ordering. *Local health centre/hospice level*, drug cupboard may not be adequate, record books may be missing, there may be staff turn over resulting in loss of know-how.

The data on barriers and blockages in morphine supply are represented in the model in Figure 1.

### Morphine regulations

Opioids are monitored by the International Narcotics Control Board which aims to promote Government compliance with international drug treaties and assist them in this effort. The National Drug Regulatory Authority operates through the Ugandan Ministry of Health, which estimates Uganda's needs each year and writes laws which cover the production of Class A drugs (WHO schedule 2) manufacture, distribution, use, and registration of all handlers. Uganda has produced model regulation guidelines [11] which are available as a model for other countries. Uganda uses morphine sulphate made up generically in-country from powder to liquid and costing between 1.8–5.6 US$ per patient per month [12]. It is stable in liquid form without refrigeration for up to three months making it especially suitable for community use.

When the scaling up of morphine use to the community was first discussed, there was suspicion from politicians, police and senior doctors who feared Uganda would become "a smugglers paradise". Therefore, the balance between encouraging use and preventing misuse was weighted towards safety. As there has not been a single case of narcotic abuse over the twelve years of hospice and three years of government use, there was a reported consensus view among Governmental and regulatory bodies that initial caution could be relaxed. This may have a significant patient impact.

"Nurses fear blame if they cannot account for all their supplies of morphine. A 24-year old man with Kaposi's sarcoma was found at home in a remote area, in great pain, with gross liver distension and dehydration. Worried about the inadequacy of pain relief it was suggested that an extra bottle (250 mls) might be left in case of breakthrough pain. The nurse prescriber was reluctant. She agreed he might soon die but argued that she might not then be able to retrieve the unused supply. The man was not seen again and was reported dead a few days later he died with prolonged convulsions. The nurse said: "Some of our clients have passed away and we can't find out where the morphine is. The previous doctor was very strict and he would check our books".

Regulation involves checking authorisation, complex book-keeping, drug storage, and occasionally caused blockages in the morphine flow (see Figure 1). Confusion arose over accreditation of NGO health professionals who had to obtain supplies through the district hospitals, as opposed to through national medical stores. One palliative care trained doctor from an NGO was refused morphine as the district hospital wanted written evidence of authority, without providing the necessary clarity about how this could be obtained. As a result a major NGO treating HIV/AIDS was without morphine. Double authorisation meant that district hospital teams had to visit two locations (MOH and medical stores) in Kampala for renewed supplies. Staff experienced difficulty in finding the appropriate person to authorise the prescription and one nurse waited 6 hours for a signature. Another had to make two journeys from up-country occupying a scarce hospital vehicle.

The regulations dictate that morphine must be double locked in a secure cupboard attached to the wall. Often pharmacy rooms were inadequate or cupboards too small to hold 500 mls bottles. Obtaining bigger cupboards was a major challenge because of different funding pathways. Drug record books could be difficult to obtain and some clinical staff found them complex to complete.

"We started to introduce morphine. They (our patients) need it. The problem is we don't have a locked cupboard......and the pharmacy is so small. It sounds easy (to put right) but they have a different administration (from the health authorities)". NGO Prescriber

"Often the pharmacist/dispenser in the hospital knew how to fill out the books however there was lack of consistency in how it was undertaken between different hospitals". Hospice Staff

New laws have been introduced allowing nurse prescribing but, until recently, doctors had to counter-sign prescriptions. The ease of achieving this depended on the doctors' attitude and much nursing time was spent chasing signatures. One nurse said that some health professionals do not see the terminally ill as a priority.

### Under-use of morphine

The patients were grateful to have been prescribed morphine which reportedly improved their quality of life, felt that it prolonged life, and had few side effects (at 5 mgs per 5 mls, the only strength available at District level). One patient said "*I cannot imagine being in that state (of pain) again". *However professional *fears *about morphine proved difficult to shift and, despite undergoing a 5-day training programme, one district hospital returned the first supplies unused.

"There are 10 doctors in the hospital and only 3 are willing (to sign prescriptions). The others you have to keep begging. I visit one ward with cancer and HIV patients and there I have to keep asking him to write but he doesn't write. He doesn't take it seriously or he doesn't think he should take it from a nurse who is lower (than he is)". Nurse Prescriber

Some nurses blamed doctors for perpetuating fears that morphine hastened death while others thought public opinion responsible. In one rural area local radio was used to re-assure people that the palliative care team "do not kill".

"In Hoima they say "when you go to that doctor you are going to die(because they give strong medicine) so we are starting to plan a radio programme maybe quarterly or monthly to talk about palliative care and try to wipe away that image that we kill". Nurse Prescriber

### Lack of essential drug and human resource shortages

Morphine should be used with other analgesics according to the WHO analgesic ladder. Use was curtailed if the district hospital ran out of simple analgesics or laxatives. Patients were asked to buy such supplies and many could not afford to do so.

Staff shortages also hindered availability. Although the HAU is well-staffed, government hospitals had severe nurse shortages. The tertiary referral hospital in Kampala with 33 wards had only one palliative care nurse to prescribe and monitor morphine (a second was in training). She covered only two wards, cancer and gynaecology, and palliative care was unavailable to many HIV/AIDS patients.

With only 200 pharmacists in the country, and 300 pharmaceutical assistants, fewer than 10 Districts in Uganda have a trained pharmacist. Nurses felt that having a pharmacist with a positive attitude to morphine use was key to obtaining uninterrupted supplies. The serious shortage of drug inspectors also weakened the functioning of the system.

### Quality of care

Quality was measured in two audits (phase 2) from rural and urban hospice sites (see Table 1). Clinical care standards in trained nurses were high although, in 7 cases, the clinical examination was considered by the observer to be incomplete (no torch examination of the mouth, no abdominal or rectal examination in the presence of constipation, no examination of the spine in suspected vertebral collapse). Validated pain measurement to titrate morphine was under-used in both audits with a reduction in completion of pain scores across successive visits. Follow up after starting morphine is important to assess adequacy of pain relief and potential side effects. Thirty-eight patients (76%) were not re-visited within the recommended 3 days, and in 25 cases (50%) the follow-up was 5 days or longer. The method of procuring further morphine was not clear in 74% of notes (n = 37) although the information may have been verbally delivered. However, prescription details were clearly written in 84% files (n = 42).

**Table 1:** Audit data: observation of nurse consultations in patients' homes (n = 21) and file reviews

	**Yes Frequency (Percent)**	**No Frequency (Percent)**	**Missing**
*Pain measurement questions*			
Was there a measure of quality of life (sleep and mobility)?	17 (81%)	4 (19%)	0
Was the pain scale (VAS) used to monitor pain?	5 (23.8%)	16 (76.2%)	0
Was duration of the pain noted?	18 (85.7%)	3 (14%)	0
*Consultation quality indicators*			
Were patient's views on morphine sought?	6 (28.6%)	15 (71.4%)	1
Were instructions clearly given?	19 (90.5%)	2 (9.5%)	1
Were side effects discussed?	14 (66.7%)	7 (33.3%)	
Was a laxative prescribed?	14 (66.7%)	5 (23.8%)	2 (9.5%)
Was the patient fully examined?	13 (61.9%)	7 (33.3%)	1
Was a follow up date arranged?	21 (100%)	0	0
Were details of the prescription written in notes?	20 (95.2%)	0	1 (4.8%)

Financial costs of illness are only one aspect. At home or in hospital, relatives have to care for the sick and this may result in the principal bread winner giving up work.

### Identifying patients and following them up

The logistics of identifying and following-up patients in rural areas are complex. With only one dedicated vehicle or a communal hospital vehicle, co-operation with community volunteers was invaluable. One rural hospice team said they could not function without them. Such liaisons ran into trouble for lack of funds to pay even expenses, let alone "incentives". If a patient lived far away, the volunteer or relative were asked to return for repeat drugs and report progress, but this incurred expense and time. Frequently, patients presented late and were not reviewed after the first morphine prescription.

### Costs to patient

Although user fees were officially banned in Uganda in 2002, charges for treatment are still in place as financial pressures are passed on. The Hospice charged patients 5,000 shillings (£2.70) per week for all care including drugs, which only one third could afford. Even when morphine is free, patients expressed distress at the costs of transport to collect it. Some patients move nearer to clinics in order to access a continuous supply of pain relieving drugs, subsequently ending their lives far from their children and families.

"If I am in Congo there is nothing there (no drugs). For 2 months in Congo I was without drugs. I spread the dose (2 week's worth) out for 2 months. I'd like to go home but can't because of the tablets. That is why I am here". Patient

"I stay about 30 miles away but I come to stay in my son's place in Kampala. The Hospice picks me up with their transport (weekly). It costs 5,000 shillings (£2.75) every week for the hospice. But the journey home costs 6,000 shillings (£3) to my village. If I can find transport money I go home. Usually once a month to look after my garden". Patient

## Discussion

From this initial evaluation of this innovative multi-agency morphine roll-out programme, we wish to disseminate 5 key lessons learned from the Ugandan experience.

First, regulation. As demand for morphine expands, adequate supply at national level must be assured by forward planning. The two functions, opioid availability and prevention of narcotic abuse, should be separate as too many legal obstacles for busy health professionals are a disincentive to use. No country has recorded diversion of therapeutic oral morphine to illegal use and many experts believe that stiff controls could be relaxed, at least at the prescriber-patient level [13]. There is no reason, if district orders match district estimates, that morphine cannot be obtained in the same way as other drugs.

Second, on-going support. It is important that the palliative care programme is owned by district health authorities. Although initial district training is costly in terms of nurse time, transportation and per diems, new money is now becoming available through the Global Fund and the President's Emergency Plan For AIDS Relief which includes funding for palliative care. Monitoring the quality of the service is important as palliative care nurses work in isolated environments. The Palliative Care Association of Uganda, under the umbrella of the new pan-African Palliative Care Association (APCA), aims to help develop and promote standards.

Third, nurse training. In Uganda it takes 9 months to become a specialist palliative care nurse (and be allowed to prescribe). Since 1993, 600 health professionals have been trained. Some question the length of training in view of the huge unmet need. But, if shortened, nurses' ability to diagnose pain accurately and prescribe effectively might be jeopardised. The Director of HAU suggests that the Ugandan training programme could be adapted to suit different health infrastructures and different types of nurses and proposes that a group of nurses and one doctor could be trained per region, who could then train others.

Fourth, the use of community volunteers. Community based volunteers are essential but lack of funding, regulation, training and support are concerns [14,15]. In Zambia, it was shown that transport costs alone accounted for 33% of home-carer costs [16]. HAU believes that without a proper palliative care component home care can be equated to home neglect [17]. The answer might be for clinical staff to lead and supervise a team of carers including traditional healers.

Fifth, the danger of health care costs displacing other essentials such as food. Forty-two percent of monthly Ugandan household expenditure is spent on food [18], and illness diverts money from this. A WHO sponsored survey in Botswana, Ethiopia, Tanzania, Zimbabwe and Uganda [19] identified practical needs of the dying and found loss of income was a huge problem for two thirds of the patients. Multi-sectoral links between a palliative care service and organisations supplying food and poverty reduction are essential to alleviate health-related family poverty.

## Conclusion

The magnitude of unnecessary suffering in countries like Uganda is so huge that pain relief should be as important a public health priority as anti-retroviral therapy, and the two closely linked. As money becomes available from the Global Fund, and as 15% of PEPFAR funding has been targeted for palliative care, this will help support other national palliative care programmes. But careful planning, education and monitoring are necessary. The Ugandan experience may be seen as a result of concerted lobbying and policy wok through HAU, which is likely to be necessary in other countries that wish to replicate their success. A recent review of palliative care in Sub-Saharan Africa found a chronic lack of African-relevant evidence on outcomes in palliative care [20,21] despite a wealth of practitioner experience. It is hoped that lessons learnt from Uganda will add to the knowledge and encourage other countries to adopt similar programmes to relieve the epidemic of silent suffering.

## Competing interests

The author(s) declare that they have no competing interests.

## Authors' contributions

DL designed the study and undertook data collection. DL and RH analysed the data and drafted the manuscript. Both authors read and approved the final manuscript.

**Table 2:** Myths and barriers to prescribing morphine

**MYTHS**	**BARRIERS**
Professional fears about safety of morphine and addiction	Logistic supply chain and transport inadequacies
Public fears that morphine expedites death	Lack of pharmacists and pharmacy support
Perceived difficulties in predicting national requirements	Lack of trained palliative care staff
Fears about illegal diversion	Over regulation, legal barriers and complex regulations
Fear of inaccurate diagnosis	Difficulties finding patients in need of care and following them up
Perception that all therapeutic morphine prescribed needs to be accounted for	Lack of hospital/palliative care vehicles
Low priority given by medical staff to the dying	Palliative care nurse training lengthy
	Cost of complimentary drugs e.g. laxatives or Step 1 and 2 analgesics
Perceptions that laws governing therapeutic morphine are difficult to change	Morphine storage difficulties
	Health staff overwhelmed
	Low priority by hospitals to palliative care

## Pre-publication history

The pre-publication history for this paper can be accessed here:


